# Recombinant Botulinum Neurotoxin Hc Subunit (BoNT Hc) and Catalytically Inactive *Clostridium botulinum* Holoproteins (ciBoNT HPs) as Vaccine Candidates for the Prevention of Botulism

**DOI:** 10.3390/toxins9090269

**Published:** 2017-09-03

**Authors:** Robert P. Webb, Theresa J. Smith, Leonard A. Smith, Patrick M. Wright, Rebecca L. Guernieri, Jennifer L. Brown, Janet C. Skerry

**Affiliations:** 1 US Army Medical Research Institute of Infectious Diseases, Fort Detrick, Frederick, MD 21702, USA; 2 Ke’aki Technologies LLC, United States Army Medical Research Institute of Infectious Diseases, Fort Detrick, Frederick, MD 21702, USA; theresa.j.smith.ctr@mail.mil (T.J.S.); jennifer.l.brown436.ctr@mail.mil (J.L.B.); janet.c.skerry.ctr@mail.mil (J.C.S.); 3 Office of the Chief Scientist, United States Army Medical Research Institute of Infectious Diseases, Fort Detrick, Frederick, MD 21702, USA; 4 Clinical Research Management, United States Army Medical Research Institute of Infectious Diseases, Fort Detrick, Frederick, MD 21707, USA; patrick.m.wright.ctr@mail.mil

**Keywords:** *Clostridium botulinum*, botulinum neurotoxin (BoNT), vaccine, catalytically inactive BoNT holoprotein (ciBoNT HP), BoNT subtype toxin, BoNT mosaic toxin

## Abstract

There are few available medical countermeasures against botulism and the discontinuation of the pentavalent botulinum toxoid vaccine by the Centers for Disease Control and Prevention in 2011 has resulted in the need for a safe and effective prophylactic alternative. Advances in genetic engineering have resulted in subsequent vaccine efforts being primarily focused on the production of highly purified recombinant protein antigens representing one or more domains of the botulinum neurotoxin. Recombinant subunit vaccines based on the carboxy one-third of the toxin (Hc) developed in our lab against serotypes A-F have been shown to be safe and effective. However, in response to the identification of an ever increasing number of BoNT subtypes with significant amino acid heterogeneity, we have developed catalytically inactive BoNT holoproteins (ciBoNT HPs) in an attempt to elicit greater protective immunity to address these toxin variants. Here we report the production of ciBoNT/B1 HP, ciBoNT/C1 HP, ciBoNT/E1 HP and ciBoNT/F1 HP and compare the immunological and protective abilities of ciBoNT HPs and BoNT/A Hc, BoNT/B Hc, BoNT/C Hc, BoNT/E Hc and BoNT/F Hc vaccines when challenged with homologous and heterologous toxins. Our results suggest the ciBoNT HP vaccines exhibit superior potency after single vaccinations but multiple vaccinations with BoNT/Hc antigens resulted in increased survival rates at the toxin challenge levels used.

## 1. Introduction

Botulinum neurotoxins (BoNTs) comprise a phylogenetically diverse group of bacterial AB protein toxins that are the causative agent of the neuroparalytic disease botulism. The BoNTs are 150 kDa proteins that contain three distinct functional domains. The enzymatic domain is contained within the 50 kDa light chain (LC) and the 100 kDa heavy chain (HC) domain is further delineated into the 50 kDa translocation domain (Hn) and 50 kDa receptor binding domain (Hc). While the intact BoNT proteins are extremely potent, independently produced domain-length fragments show little or no toxicity when injected into animals [1,2].

While recorded incidents of botulism have been noted for hundreds of years, the disease was recognized as a public health concern in the United States after 32 people died in five outbreaks associated with ripe olives in 1919 and 1920 [3]. These incidents emphasized the need for effective treatments, such as antitoxins and vaccines, to counteract human and animal botulism. Early vaccines were developed to provide a more humane way of immunizing animals for the production of equine-based antitoxins to be used as human medical therapeutics, and also to protect livestock from botulism. These vaccines were produced by inactivating the toxin proteins through exposure to liquid formaldehyde, a procedure known as “toxoiding”.

The discovery prior to and during the second World War that foreign powers were either using, or considering the use, of botulinum toxin as a weapon provided an impetus for the development of vaccines as countermeasures to protect armed forces from intoxication [4]. Initial efforts by the U. S. Army produced a bivalent AB toxoid vaccine that was found to be effective, but generated undesirable reactions due to the crude nature of the vaccine [5]. Further vaccine research was conducted in the 1950s and 1960s involving the optimization of bacterial cell growth, toxin production and purification, effective toxoiding techniques, and studies of the effects of alum-based adjuvants to boost the immune response. Monovalent, bivalent, and pentavalent toxoids were developed and tested in man and various animals [6]. This information was used by Parke Davis Co. and the Michigan Department of Health to produce a pentavalent botulinum ABCDE toxoid vaccine (PBT). The PBT vaccine was administered under CDC Investigational New Drug-161 (IND-161) to at-risk workers for exposure to botulinum toxins as well as the U.S. Army Office of Surgeon General (IND 3723) for use with military personnel at-risk during deployment [7]. However, the CDC announced that it would no longer administer the PBT after November 2011 due to declining potency in the aging stocks [8].

The ability to produce specific genetic sequences that could be mobilized into heterologous microbial expression platforms to produce recombinant proteins that could be purified to homogeneity came with the advent of modern molecular biology techniques. This enabled the production of pure vaccine antigens that retained the immunogenicity of the parent proteins, but were devoid of toxicity. It was no longer necessary to subject protein antigens to harsh chemical treatments that abolished toxicity but could also lead to physical changes resulting in diminished immunogenicity [9,10].

The development of genetically constructed clostridial vaccines utilized a subunit approach where protein fragments representing the individual nontoxic functional domains were produced and assessed for their ability to elicit protective immunity. Initial subunit vaccines that were tested involved tetanus protein fragments [11,12,13]. The tetanus toxin protein is similar to the botulinum neurotoxin in that it is a neurotoxin of approximately the same size, it contains the same three structural domains, and it shares 34–41% identity with BoNTs at the amino acid level; which is similar to differences among the individual BoNT serotypes. Demonstration of the efficacy of the tetanus subunit vaccines prompted development of botulinum subunit vaccines using BoNT Hc domains. While tetanus toxin is a single protein, multiple serologically distinct botulinum toxins have been identified. There are currently seven BoNT serotypes, denoted A–G, that differ by 37.2–69.6% at the amino acid level, which requires the development of seven individual serotype-specific vaccines for full spectrum prophylaxis. Within each serotype, with the exception of BoNT/G, there are two to eight BoNT subtypes that provide additional variability, with amino acid differences ranging from 1.6 to 36.2% [14]. This heterogeneity impacts binding and protection with neutralizing antibodies and there are concerns that they may prove problematic to the development of prophylactic and therapeutic agents developed against a dissimilar subtype [15].

BoNT Hc vaccines for all seven serotypes have been developed in our laboratory and others [16], and testing has also been done comparing BoNT/A Hc, BoNT/B Hc, and BoNT/C Hc in monovalent and trivalent formats [17,18]. In each of the described studies, the vaccines are based on sequences from single toxin subtypes (A1, B1, C1, D/C, E1, F1, and G). The BoNT Hc vaccines have been demonstrated to be highly effective vaccines, providing complete protection against significant challenge levels of parental (homologous) toxin, and they are safe and relatively easy to produce in a scalable microbial expression platform [7,19]. In addition, a recombinant bivalent BoNT/A1 and /B1 Hc subunit vaccine (rBV A/B) originally developed at USAMRIID has successfully completed a phase 2 clinical study [2]. While these vaccines are effective immunogens against their homologous toxins, their effectiveness against heterologous toxins has not been characterized.

Advances in rapid gene synthesis and targeted mutagenesis techniques have enabled the production of complete BoNT holoproteins with mutations designed to render the protein nontoxic. Several of these nontoxic holoproteins have been investigated for use in trafficking studies [20], as possible targeting and delivery vehicles for drugs [21,22], and as vaccines [23,24,25,26]. We previously reported on the production and immunological assessment of a recombinant, catalytically inactive BoNT/A1 holoprotein vaccine (ciBoNT/A1 HP) which was found to have greater potency than recombinant protein antigens representing any other /A1 subtype domain, or combination of domains, against a challenge of both parental toxin and two dissimilar toxin subtypes (Webb 2009). Here we expand upon this line of research and report the production of recombinant ciBoNT/B1 HP, ciBoNT/C1 HP, ciBoNT/E1 HP and ciBoNT/F1 HP protein antigens and the evaluation of these vaccines for potency, stability, and efficacy in both monovalent and multivalent formulations, including comparative studies with BoNT Hc vaccines.

## 2. Results and Discussion

### 2.1. Production and Purification of Recombinant ciBoNT/B1 HP, ciBoNT/C1 HP, ciBoNT/E1 HP, and ciBoNT/F1 HP

**Figure 1:**
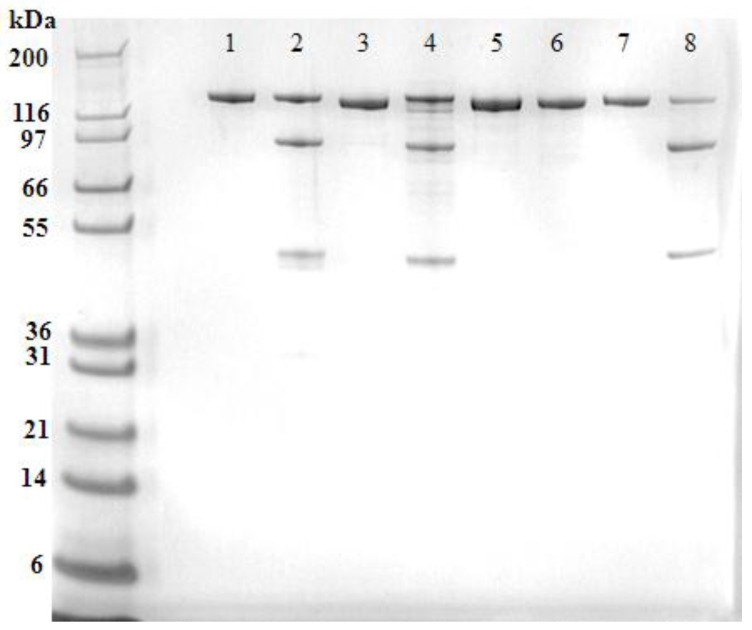
SDS-PAGE gel of approximately 1 µg of purified, recombinant ciBoNT/B1 HP, ciBoNT/C1 HP, ciBoNT/E1 HP, and ciBoNT/F1 HP. Left lane-Mark 12 molecular weight marker, lane 1-ciBoNT/B1 HP, lane 2-BoNT/B1 toxin, lane 3-ciBoNT/C1 HP, lane 4-BoNT/C1 toxin, lane 5-ciBoNT/E1-6xHis HP, lane 6-BoNT/E3 toxin, lane 7-ciBoNT/F1 HP, lane 8-BoNT/F1 toxin.

The recombinant ciBoNT/B1 HP, ciBoNT/C1 HP, ciBoNT/E1 HP, and ciBoNT/F1 HP were purified to a homogeneity of >97% as determined by SDS-PAGE analysis (Figure 1). The recombinant ciBoNT HPs were produced almost exclusively as a single chain molecule, most likely due to the lack of bacterial or host enzymatic cleavage into the dichain form commonly observed in the processed toxin.

**Figure 2:**
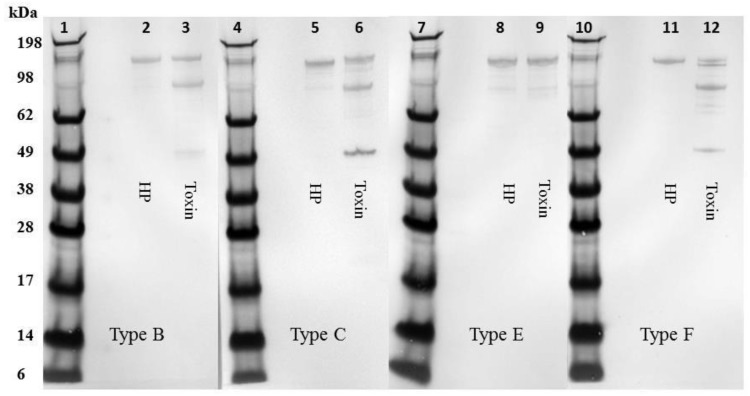
Western blot analysis of the approximately 1 µg of purified, recombinant ciBoNT/B1 HP, ciBoNT/C1 HP, ciBoNT/E1 HP, and ciBoNT/F1 HP. Lanes 1, 4, 7, 10-SeeBlue Plus 2 protein standard, lane 2-ciBoNT/B1 HP, lane 3-BoNT/B1 toxin, lane 5-ciBoNT/C1 HP, lane 6-BoNT/C1 toxin, lane 8-ciBoNT/E1-6xHis HP, lane 9-BoNT/E3 toxin, lane 11-ciBoNT/F1 HP, lane 12-BoNT/F1 toxin. The primary antibodies were equine polyclonal antitoxins used at a final concentration of 0.05 IU/mL for each serotype.

Western blot analysis of the recombinant ciBoNT HPs (Figure 2) confirmed the homogeneity of the recombinant protein and revealed some minor cleavage of the single chain protein into the dichain form in the ciBoNT/C1 HP and ciBoNT/E1 HP. Because neither the native toxins nor the ciBoNT HPs shown in Figure 1 and Figure 2 were trypsinized, this limited cleavage to the dichain form is attributed to Picha enzymes at some point during the purification process.

### 2.2. Potency of ciBoNT HP and BoNT Hc Vaccines against Homologous Toxins

Potency assays provide sensitive quantification of the neutralizing ability of various BoNT Hc and ciBoNT HP vaccines. Our assays involve vaccination of mice with decreasing antigen concentrations followed by a standard intraperitoneal (IP) toxin challenge level of 1000 LD_50_ per mouse. The potencies use a single vaccination followed by toxin challenge 21 days later, with the exception of the BoNT/E Hc assay, which requires two vaccinations at 0 and 14 days with toxin challenge at 35 days (21 days after the final vaccination). The results are used to calculate a theoretical vaccine concentration that will protect 50% of animals from intoxication at the same challenge dose (ED_50_). With these assays, lower ED_50_ values indicate more potent vaccines that require smaller amounts of the vaccine antigen to elicit protective immunity. These potency assays were employed to evaluate the protective immunity elicited by both the BoNT Hc and ciBoNT HP vaccines in both monovalent and multivalent formats.

Protection with monovalent ciBoNT HP vaccines was excellent, with initial ED_50_ values ranging from 8 to 11 ng for ciBoNT/A1 HP, ciBoNT/C1 HP, and ciBoNT/F1 HP, and 49 ng for ciBoNT/E1 HP (Table 1). This was 3–15 times better compared to corresponding ED_50_ values with their monovalent BoNT Hc counterparts. The ED_50_ values for the monovalent versus multivalent ciBoNT/ACEF HP vaccines showed equivalent potency, except BoNT/E, where the monovalent format is marginally more potent. The BoNT/A1 Hc, BoNT/B1 Hc, and BoNT/C1 Hc vaccine formulations also show equivalent potencies in both monovalent and polyvalent formulations, with less than two-fold difference in ED_50_ values (Table 1). However, the polyvalent BoNT/E1 Hc and BoNT/F1 Hc vaccines have ED_50_ values that are approximately 7-fold and 4-fold higher (less effective), respectively, than their counterparts in the pentavalent Hc vaccine, indicating there is some loss of potency in the multivalent formations when compared to monovalent vaccines.

The stability of these vaccines was also assessed by comparing potency assay results for the ciBoNT HP and BoNT Hc vaccines at 6 months after formulation (Table 2). Monovalent and multivalent vaccines were formulated with aluminum hydroxide adjuvants and stored at 2–8 °C for the time course of the vaccinations. The ciBoNT HP vaccines showed good potency and stability in both monovalent and polyvalent formulations. Overall, ED_50_ values ranged from 6 to 21 ng regardless of serotype or formulation with the exception of the ciBoNT/E HP vaccine, where results ranged from 129 to 132 ng (Table 2). There were no statistically significant differences within serotype between the ciBoNT HP monovalent and multivalent formulations, or between 0 and 6 months, indicating that these vaccines are highly potent, stable, and amenable to multivalent formulation.

The monovalent BoNT Hc vaccine 6 month potency ED_50_ values were slightly higher than their ciBoNT HP counterparts, with a range of 19–201 ng, regardless of serotype, using a two vaccination protocol for BoNT/E Hc (Table 2). However, the multivalent formulations displayed broader variations in ED_50_, with values ranging from 14 ng to 969 µg. The BoNT Hc monovalent vaccines showed good stability, with no significant differences seen between the initial and 6-month potencies. In addition, there were no significant differences between formulations within each serotype with the BoNT/A1 Hc, BoNT/B1 Hc, and BoNT/C1 Hc vaccines. However, there were significant differences between the ED_50_ values of monovalent and multivalent formulations of the BoNT/E1 Hc and BoNT/F1 Hc vaccines, with a higher (less effective) potency seen in these vaccines when they are combined.

### 2.3. Comparative Potency of ciBoNT HP and BoNT Hc Vaccines against Heterologous Toxins

Comparative potency studies between the ciBoNT HP and BoNT Hc vaccines resulted in more dramatic differences when these vaccines were challenged with heterologous toxin subtypes.

The ciBoNT/A HP and BoNT/A Hc vaccines were comparatively assessed against BoNT/A1, BoNT/A2, and BoNT/A3 toxins as reported in a previous study [25]. Toxin subtypes BoNT/A2 and BoNT/A3 diverge from the parental BoNT/A1 amino acid sequence by 10.1% and 15.4%, respectively (Table 3).

Despite these differences, a single vaccination with ciBoNT/A HP provides effective protection against these toxins, with ED_50_ values of 18 ng against BoNT/A1 and 132 ng and 144 ng against subtypes BoNT/A2 and BoNT/A3, respectively, representing less than 10-fold variation in protection across all toxin challenges (Table 4A).

The BoNT/A1 Hc vaccine elicited an ED_50_ of 52 ng against the parental toxin but produced comparatively poor potency values of 5.9 µg and 18 µg against the BoNT/A2 and /A3 subtypes, respectively; these represent differences of over 100-fold and almost 400-fold (Table 4A).

The ciBoNT/B1 HP and Hc vaccines were also assessed against both the parental toxin and two dissimilar subtypes. The ciBoNT/B1 HP elicited excellent protective immunity with ED_50_ values of 19, 67, and 32 ng against challenges of BoNT/B1, BoNT/B2, and BoNT/B4, respectively (Table 4B). The BoNT/B Hc vaccine potency assay resulted in an ED_50_ of 33 ng against BoNT/B1 but demonstrated poor potency against BoNT/B2 with an ED_50_ of 24 µg, which is a 700-fold decrease in potency. The ED_50_ against BoNT/B4 was 77 µg, suggesting very poor potency (Table 4B). While the overall differences in amino acid sequence between BoNT/B1 and BoNT/B2 or BoNT/B4 are only 4.4% and 6.8%, respectively, the variations within their Hc regions is higher, at 9% and 11%, respectively (Table 3). This may partly explain the relatively poor performance of the BoNT/B Hc vaccine against these toxins. However, it is important to remember that while heterogeneity in the subtypes correlates with poorer ED_50_ values in the Hc vaccines, the actual potency values may also be influenced by the percentage of amino acid changes that are located within epitopes responsible for the production of neutralizing antibodies.

The ciBoNT/C HP provided excellent protection against BoNT/C1 and BoNT/CD, with ED_50_ values of 15 and 27 ng, respectively (Table 4C). The latter potency value was unexpected, given an overall difference of 24% in amino acid sequence between BoNT/C1 and BoNT/CD (Table 3). However, while the Hc portions of these toxins differ by 63%, the LC and Hn portions show 95% identity, which may provide an immunological advantage for the additional epitopes contributed by the ciBoNT/C1 HP vaccine. No protection was seen against BoNT/DC, which shows greater variability in the LC-Hn than the Hc region and has an overall difference of 35% from BoNT/C1 (Table 3). The BoNT/C1 Hc vaccine also provided good protection against BoNT/C1, but no discernable protection against either BoNT/CD or BoNT/DC. This was anticipated, as there is a 63% difference in amino acid sequence between the Hc of BoNT/C1 and BoNT/CD, and with BoNT/DC the Hc proteins differ by 24% (Table 3).

In contrast to other BoNT Hc serotype potency bioassays which employ a single administration of antigen, the BoNT/E Hc potency assay has historically required two vaccinations to produce analyzable sigmoid survival curves [27]. Therefore, potency comparisons were initially done using a two-vaccination protocol with both the ciBoNT/E HP and BoNT/E Hc. Results for ciBoNT/E HP with the two-vaccination protocol showed equivalent potencies to those with other ciBoNT serotypes, with ED_50_ of 4–22 ng. When the ciBoNT/E1 HP was applied in a single vaccination protocol, it still elicited ED_50_ values substantially lower than the corresponding BoNT/E Hc, with ED_50_ ranging from 62 to 160 ng (Table 4D). Both ciBoNT/E HP potency protocols resulted in good to excellent protection against all three challenge toxin subtypes. However, the BoNT/E Hc vaccines provided poor protection, despite using the two-vaccination protocol. All ED_50_ were in the microgram range, and there was no effective potency seen with BoNT/E Hc challenged with BoNT/E4. This result was unexpected, given that the Hc of BoNT/E4 differs by only 2% from the Hc of BoNT/E1 (Table 3). In fact, these three toxins differ by less than 3% in amino acid sequence both overall and in their Hc regions, which are reflected in their generally equivalent potency results. The reasons for the overall poorer performance of the BoNT/E vaccines, particularly the BoNT/E Hc vaccine, when compared with the other serotypes are not yet understood.

A highly abbreviated study was performed with the ciBoNT/F1 HP which was assessed in potency studies against the parental BoNT/F1 and BoNT/F7 subtype. No corresponding BoNT/F Hc study was performed. While excellent protection is seen against BoNT/F1, with an ED_50_ of 2 ng, the ED_50_ against BoNT/F7 is 1.1 µg. This was not unexpected, as these two toxin types show a 26% overall difference in amino acid sequence (Table 3).

### 2.4. Efficacy of ciBoNT HP and BoNT Hc Vaccines against Heterologous Toxins

In order to assess the cumulative effect of multiple vaccinations on protection against both homologous and heterologous BoNT toxin challenges, a series of efficacy studies were performed by vaccinating mice one, two, or three times (1X, 2X, 3X) with 1 µg of antigen per animal at four-week intervals, with toxin challenges of 1000 mouse IP LD_50_ two weeks after the final vaccination. Animals were vaccinated with ciBoNT/A1 HP, ciBoNT/B1 HP, ciBoNT/C1 HP or BoNT/A1 Hc, BoNT/B1 Hc, BoNT/C1 Hc vaccines and challenged with both homologous and heterologous toxins.

The BoNT/A2 and BoNT/A3 challenge results after a single vaccination are lower than expected when compared to potency scores (Table 5A). The potency results predicted that complete protection against all three toxins should be achieved with a single vaccination of ciBoNT/A HP, but only 50% survival was seen against BoNT/A2 and with BoNT/A3 survival was only 30%. Similarly, with BoNT/A1 Hc against BoNT/A1 protection was incomplete, and survival was poor against BoNT/A2 and /A3.

As a rule, peak antibody levels are typically achieved approximately three weeks after exposure or single vaccination with a protein antigen. It should be noted that the single dose efficacy assays are challenged at two weeks post vaccination while the single dose potency challenges are performed at three weeks post vaccination, and apparently with BoNT/A antibodies, the additional week is needed to develop sufficient high affinity neutralizing antibodies to enable complete protection against BoNT/A2 and BoNT/A3. However, there is complete to near complete survival after two vaccinations and challenge with all three toxins, indicating that both vaccines are effective against homologous and heterologous BoNT/A subtypes after multiple vaccinations (Table 5A).

With BoNT/B, sufficient neutralizing antibodies to enable complete protection against toxin challenge appear to be produced earlier than with BoNT/A, so that BoNT/B efficacy results reflect potency results, with complete protection against all three BoNT/B subtypes after a single vaccination with ciBoNT/B1 HP and against BoNT/B1 after a single vaccination with BoNT/B1 Hc. Protection improves against BoNT/B2 and BoNT/B4 after multiple vaccinations with BoNT/B1 Hc, but complete protection is not achieved even after 3 vaccinations (Table 5B). This might be attributed to variances in the amino acid sequence of the Hc region of these toxins, which are 8.4% with BoNT/B2 and 10.5% with BoNT/B4 (Table 3).

The ciBoNT/C HP vaccine provides excellent protection against BoNT/C1 and BoNT/CD, with a single vaccination and almost complete protection against BoNT/DC after 2 vaccinations. The BoNT/C Hc vaccine provided complete protection against the parental toxin after a single vaccination. However, the BoNT/C Hc vaccine provided poor protection against the BoNT/CD and /DC mosaic toxins; providing only 30% and 70% protection, respectively, after 3 immunizations (Table 5C). The immunological challenges experienced by the BoNT/C Hc vaccine are most likely due to very significant sequence differences among the mosaic CD and DC toxins (Table 3).

### 2.5. Toxicity Bioassay

The results of the mouse toxicity bioassay are summarized in Table 6. All production lots of the ciBoNT protein antigens are initially tested for residual toxicity at a minimum level of 5 µg and if no deaths are observed, higher doses of the recombinant ciBoNTs are investigated.

All of the ciBoNTs were found to be completely non-toxic at the 5 µg dose except for the ciBoNT/E1. We are currently assessing recombinant ciBoNT/E1 proteins with alternative or additional mutations designed to reduce residual toxicity, but maintain comparable potency values. The ciBoNT/A1 was found to be non-toxic at 50 µg and the ciBoNT/B1 and ciBoNT/F1 at 25 µg. The ciBoNT/C1 displayed some residual toxic effects at the 25 µg dosage. The ciBoNTs do display some toxicity at higher concentrations, but at doses that are roughly three magnitudes of order greater than the actual therapeutic dose as determined by the ED_50_.

## 3. Discussion

Collectively, the results from comparative potency and efficacy studies between these two vaccine candidates suggest that the ciBoNT HP vaccine antigens elicit a more robust neutralizing antibody response that provides better protection against a challenge from the parental toxins and superior protection against challenges from dissimilar subtypes. Protection using ciBoNT HP in monovalent and multivalent formulations against homologous toxins is excellent, with potency ED_50_ values for ciBoNT/A1 HP, ciBoNT/B1 HP, ciBoNT/C1 HP, and ciBoNT/F1 HP ranging from 6 to 21 ng. Protection with ciBoNT/E HP is marginally less effective, with potency ED_50_ values of 49–139 ng. The poorer performance of the ciBoNT/E HP and BoNT/E Hc vaccines relative to the other serotypes might be partly explained by differences in the structure of the toxin proteins. It is known that BoNT/A and BoNT/B adopt a planar “butterfly-like” conformation, where the catalytic and receptor-binding domains are found on opposite sides of the translocation domain, but BoNT/E has a more compact, globular “shoe-like” conformation with interactions seen among all three functional domains [28,29]. These interactions are mainly hydrophobic in nature. It is thought that the BoNT/E conformation may enable faster translocation across endosomes, and that this may be why BoNT/E is faster acting than BoNT/A or BoNT/B [30]. The majority of neutralizing antibodies are known to be produced against epitopes composed of one or more peptide loops exposed to the aqueous environment, and the compact nature of BoNT/E may result in epitope structures that are less accessible for immune responses, which could hamper the generation of effective neutralizing antibodies. In addition, the BoNT/E Hc proteins contain hydrophobic regions, which might result in the formation of dimers. These dimer molecules could mask certain antibody epitopes that might be exposed with other toxin serotypes. Thus, differences in the ability of ciBoNT/E HP and BoNT/E Hc proteins to elicit neutralizing antibodies might be traced to their unusual structure. The BoNT/F Hc vaccine also displays less effective potency than other serotypes. This may also be the result of protein structural differences versus other serotypes; however, detailed structures for this toxin type are not yet available for examination.

The individual monovalent BoNT Hc vaccine and the multivalent BoNT/ABCEF Hc vaccine ED_50_ values indicate they have a slightly lower (worse) potency than their ciBoNT HP vaccine counterparts when challenged with BoNT/A1, BoNT/B1, and BoNT/ C1; ED_50_ values range from 19 to 222 ng, with decreasing (better) potency seen when tested after 6 months storage. In contrast, the BoNT/E1 Hc and BoNT/F1 Hc vaccine components in the polyvalent formulation displayed significantly lower potency values (6.9-fold and 2.9-fold respectively), as compared to the monovalent formulation potency values. The ciBoNT/ACEF HP vaccine did not display an analogous loss of potency in the ciBoNT/E1 HP and ciBoNT/F1 HP components, and the monovalent and polyvalent formulations at 0 and 6 months displayed consistent protection against challenges with their corresponding parental toxins, suggesting the holoproteins might be more amenable to polyvalent formulation than their Hc subunit counterparts.

Comparative potency assessments against heterologous toxin subtypes were performed using monovalent ciBoNT/A1 HP, ciBoNT/B1 HP, ciBoNT/C1 HP, ciBoNT/E1 HP, ciBoNT/F1 HP and their BoNT Hc counterparts. The potency values generally reflect divergent amino acid sequences observed in the subtypes, with lower (better) ED_50_ values observed among the ciBoNT HP vaccines than with the BoNT Hc vaccines when challenged with heterologous toxins. BoNT/E1 Hc vaccines showed poor protection against both homologous and heterologous toxins in these assays, with an ED_50_ of 1.13 µg against BoNT/E1, 2.42 µg against BoNT/E3, and >10 µg against BoNT/E4. Results from the monovalent/multivalent potency studies showed a slightly better potency of 969 ng–1.53 µg versus BoNT/E3. This slight difference in performance of the BoNT/E Hc vaccines may be attributed to differences in their storage buffers. The BoNT/E Hc used in the BoNT/E1, /E3, /E4 toxin comparisons was produced and stored in 15 mM succinate, pH 4.0, while the BoNT/Hc in the monovalent/multivalent studies was stored in 25 mM succinate, 15 mM sodium phosphate, pH 5.0 with 5% trehalose included as an excipient. The latter formulation may have afforded increased protein stability.

The efficacy bioassays demonstrated that multiple vaccinations with ciBoNT/A1 HP, ciBoNT/B1 HP, and ciBoNT/C1 HP antigens result in complete/near complete protection against challenge with both homologous and heterologous toxin subtypes, often with a single vaccination. The analogous BoNT Hc vaccines did not show similar levels of protection until administration of 2 or 3 vaccinations and even then, none provided complete protection against any of the dissimilar subtypes. The BoNT Hc vaccines were particularly ineffective against the BoNT/DC and BoNT/CD subtypes, which might be attributed to the highly divergent nature of those particular mosaic toxins.

The immunological advantages of the recombinant ciBoNT HP vaccine antigens are most likely due to the inclusion of all three protein domains, which provides a greater diversity of potential neutralizing epitopes than the individual subunit vaccines. In addition, the ciBoNT HP proteins are most likely more structurally representative of the native BoNTs than the individual subunit vaccines, so that they would be expected to result in higher affinity antibodies to the toxins. However, despite the potential advantages, few laboratories have reported on the protective efficacy of full length, inactivated botulinum toxin proteins as vaccines against homologous toxins [23,24], and there are even fewer published studies in which full length ciBoNT HP vaccines or subunit BoNT Hc vaccines have been evaluated for stability or assessed against multiple toxin subtypes [25,31]. This may be an important gap in information, since there are currently over 40 identified toxin subtypes which exhibit amino acid differences of up to 36% within their serotype. Because the majority of the current vaccine antigens are based on one single parental toxin sequence per serotype, this variability presents challenges for the development of effective treatment of botulism as well as potential protection through vaccination.

However, the data produced in this study strongly indicates that the ciBoNT HP may offer a more robust immunological response that elicits comparatively greater potency than the BoNT Hc vaccines, particularly when challenges are performed with dissimilar toxin subtypes. Additional studies to include comparative efficacy studies with the ciBoNT/E1 HP and /E1 Hc vaccines, and potency and efficacy studies with the ciBoNT/F1 and /F1 Hc vaccines against at least two dissimilar subtypes would be helpful to provide a complete picture of the capabilities of these vaccines.

## 4. Materials and Methods

For the production of ciBoNT/HP, synthetic open reading frames (ORFs) were synthesized with a *Pichia pastoris* alcohol oxidase 1 (*AOX1*) gene codon bias. Each ORF was designed with three mutations within the conserved HEXXH catalytic active site of the BoNT LC, replacing the two histidines and single glutamic acid residues with alanine residues (ciBoNT/B1 H^230^A, E^231^A, H^234^A; ciBoNT/C1 H^229^A, E^230^A, H^233^A; ciBoNT/E1 H^212^A, E^213^A, H^216^A; ciBoNT/F1 H^227^A, E^228^A, H^231^A). The ciBoNT HP ORFs were inserted into the yeast cytosolic expression plasmid vector pPICZA and mobilized into *P. pastoris* strain X-33 (Invitrogen, Carlsbad, CA, USA) as per manufacturer’s instructions. Production strains were identified and produced as described previously [27,31]. All recombinant DNA work was done with the approval of the USAMRIID Institutional Biosafety Committee. Both the synthetic DNA ORFs and derived recombinant proteins for ciBoNT/A1 HP, ciBoNT/B1 HP, ciBoNT/C1 HP, ciBoNT/E1 HP, and ciBoNT/F1 HP have been granted exemptions from select agent status by the Centers for Disease Control and Prevention.

### 4.1. Protein Purification

The purification and characterization of the ciBoNT/A1 HP [25], and BoNT/A Hc [32], BoNT/B Hc [33], BoNT/C Hc [31], BoNT/E Hc [27] and BoNT/F Hc [34] vaccine antigens have been described previously. Robust de novo purification protocols were developed to purify recombinant ciBoNT/B1 HP, ciBoNT/C1 HP, ciBoNT/E HP, and ciBoNT/F1 HP antigens from transgenic production strains of *P. pastoris*. All chromatography separations were performed on an AKTA Purifier 900 FPLC system using Unicorn (v5.31) software.

### 4.2. ciBoNT/B HP.

Frozen *P. pastoris* paste at 1 g of paste per 20 mL of buffer was re-suspended in 20 mM sodium acetate, pH 6.5 plus 1 mM EDTA, lysed with a Microfluidics 110 S microfluidizer (Microfluidics, Westwood, MA, USA) at 20,000 psi and the crude lysate was clarified by centrifugation at 27,000× *g*. The clarified supernatant was diluted with breaking buffer containing 2.4 M ammonium sulfate to a final conductivity of 130 mS and loaded onto a GE Life Sciences Phenyl Sepharose HP column (GE Healthcare Biosciences, Pittsburgh, PA, USA) pre-equilibrated with breaking buffer plus 1.2 M ammonium sulfate. After the column was washed, the target protein was eluted by linear gradient with breaking buffer and the target protein began to elute at 95 mS. The pooled fractions with the greatest amount of the target protein were dialyzed into 20 mM sodium acetate, pH 5.5 until the conductivity reached 12 mS and the pH was adjusted to 5.3 with 20 mM sodium acetate, pH 4.5. The dialyzed pool was loaded onto a GE Life Sciences SP-HP Sepharose column pre-equilibrated with 20 mM sodium acetate, pH 5.3 containing 1 mM EDTA, washed and a linear gradient was developed with equilibration buffer plus 1 M sodium chloride. At approximately 23 mS conductivity, the target protein eluted off the column and fractions of highly purified ciBoNT/B HP were collected and dialyzed into final vaccine formulation buffer consisting of 20 mM sodium succinate pH 5.8, 15 mM sodium phosphate and 5% trehalose.

### 4.3. ciBoNT/C HP.

Frozen *Pichia pastoris* paste harboring the recombinant ciBoNT/C HP was re-suspended in 25 mM Sodium acetate, pH 6.5 containing 2 mM EDTA was lysed and clarified as described previously. The supernatant was further clarified through a 0.45 µM PVDF membrane and diluted with 2.4 M ammonium sulfate to a conductivity of 125 mS. The diluted extract was loaded onto a GE Life Sciences Phenyl Sepharose HP column pre-equilibrated with breaking buffer plus 1 M ammonium sulfate, washed and a linear gradient was developed to 100% breaking buffer which eluted the target protein. At a conductivity of approximately 70 mS, the protein began eluting off the column and pooled fractions containing the target protein were dialyzed against 25 mM sodium acetate, pH 6.0 until the conductivity reached 15 mS. This pool was then loaded onto a GE Life Sciences SP-FF Sepharose column equilibrated with 25 mM sodium acetate, pH, 5.8 and 1 mM EDTA and a linear gradient was developed with running buffer plus 1 M sodium chloride. At approximately 28 mS conductivity, the target protein eluted off the column in a single peak. Fractions containing the target protein were pooled, dialyzed as above and loaded onto a GE Life Sciences SP-HP column, eluted by linear gradient with buffer containing 1 M sodium chloride. Again, at approximately 28 mS conductivity, highly purified holoprotein eluted off the column and was then dialyzed into final formulation buffer described previously.

### 4.4. ciBoNT/E HP-6xHis.

Frozen *Pichia pastoris* cell mass harboring the target protein was re-suspended in 50 mM Tris-HCL, pH 7.8 containing 25 mM imidazole, 300 mM sodium chloride at 20 mL of buffer per gram of cell paste and lysed and clarified as described previously. The clarified supernatant was loaded onto a GE Life Sciences Nickel Sepharose column (GE Healthcare Biosciences, Pittsburgh PA) pre-equilibrated in breaking buffer, washed with 25% of elution buffer, (breaking buffer plus 250 mM imidazole) and the target protein was eluted with a linear gradient from 25% to 100% of elution buffer. Fractions containing the ciBoNT/E HP-6xHis were pooled and diluted in 20 mM sodium acetate, pH 6.4, containing 1 mM EDTA and 2.4 M ammonium sulfate, to a conductivity of 130 mS and loaded onto a GE Life Sciences Phenyl Sepharose HP column pre-equilibrated with 20 mM sodium acetate, pH 6.4 with 1.2 M ammonium sulfate. After washing to remove impurities, the target protein was eluted by linear gradient with equilibration buffer without ammonium sulfate. At approximately 75 mS conductivity, the ciBoNT/E-6X His HP eluted sharply and fractions of highly purified protein were collected and dialyzed into final vaccine formulation buffer described previously.

### 4.5. ciBoNT/F HP.

Frozen *P. pastoris* cell paste containing recombinant ciBoNT/F HP was re-suspended in 50 mM Tris-HCl buffer, pH 7.5 containing 1 mM EDTA was lysed and clarified as described previously. The supernatant was loaded onto a GE Life Sciences Q-FF Sepharose column pre-equilibrated with breaking buffer and the target protein was eluted by linear gradient with equilibration buffer plus 1 M sodium chloride. At approximately 14 mS, the target protein begins to elute off the column and pooled fractions were diluted with 20 mM sodium acetate containing 2.4 M ammonium sulfate to a conductivity of 135 mS and loaded onto a GE Life Sciences Phenyl HP Sepharose column pre-equlibrated with 20 mM sodium acetate pH 6.5 containing 1.2 M ammonium sulfate. Elution of the target protein was achieved using a linear gradient with equilibrating buffer containing no salt. At approximately 125 mS the target protein began to elute and was collected. Fractions containing the highest concentration of ciBoNT/F HP were pooled and dialyzed against 20 mM sodium acetate, pH 5.3 to a conductivity of 10 mS. This pool was loaded onto a GE Life Sciences SP-HP Sepharose column pre-equilibrated with dialysis buffer. After washing off unbound impurities the target protein was eluted by a linear gradient containing equilibration buffer plus 1 M sodium chloride. At approximately 27 mS the mostly purified protein eluted off the column and collected in fractions. This pool was again diluted to 10 mS and loaded onto a GE Life Sciences MonoS column pre-equilibrated as was the SP-HP column, and eluted in the same manner. Highly purified target was collected and dialyzed into final formulation buffer described previously.

### 4.6. Toxin Preparations

Botulinum neurotoxins complexed with nontoxic accessory proteins were purchased from Metabiologics (Madison, WI, USA) and WAKO (Kyoto, Japan), or produced at USAMRIID. Toxins produced in house were generated in 8 liter batches incubated at either 30 °C or 35 °C for 4–5 days. Supernatants from BoNT/A3, BoNT/B2, and BoNT/E4 were acid precipitated in bulk with reconstitution in PBS, pH 7.0. BoNT/F7 was produced in dialysis tubing, which was clarified by centrifugation. Supernatant from the cultures was ammonium sulfate precipitated and dialyzed into PBS, pH 7.0. Table 7 lists characteristics from the toxins used in these studies.

### 4.7. Mouse Potency Bioassays

Seven groups of 10 mice each were given intramuscular (IM) injections with dilutions of the individual antigen in 20 mM sodium succinate pH 5.8, 15 mM sodium phosphate, 5% trehalose adsorbed to 0.2% aluminum hydroxide adjuvant (Alhydrogel, Biosector, Denmark). The potencies utilized a three-fold dilution scheme ranging from 11 ng to 8.1 µg for BoNT Hc and approximately 1.2 ng–1.0 µg for ciBoNT HP. For the BoNT/E Hc potency assays, mice were given two vaccinations two weeks apart. Three weeks post-vaccination, the mice were challenged intraperitoneally (IP) with 1000 LD_50_ of either the appropriate toxin. Mice were observed twice daily for 5 days and survival results were subjected to probit analysis (SPSS). The ED_50_ (theoretical effective dose protecting 50% of mice) plus 95% confidence values were generated and used to compare potencies of the vaccines.

### 4.8. Efficacy Studies

The ciBoNT/A HP, ciBoNT/B HP, and ciBoNT/C HP antigens and the corresponding Hc antigens were compared using a mouse efficacy bioassay in which mice were given one, two or three IM vaccinations with 1 µg of antigen at 4-week intervals followed by IP challenge with 1000 LD_50_ of appropriate toxin two weeks post-vaccination. Animals were observed for clinical signs of botulism and survival was assessed at 5 days post-challenge.

### 4.9. Toxicity Bioassay

In order to assess any residual toxicity in the ciBoNT vaccine protein antigens before their inclusion in potency and efficacy assays, a preliminary toxicity assay is performed. The vaccine candidate is diluted to a concentration of 10 µg/mL in gel phosphate buffer, pH 6.2. A group of five mice are injected IP with a volume of 0.5 mL; delivering a dose of 5 µg. The mice are monitored for signs of intoxication for 15 days post-injection. If the subjects show no signs of toxicity at the 5 µg dose, independent assays may be performed using a dose of 25, 50 or 100 µg to determine the upper level of toxicity. If any of the ciBoNT proteins are found to have any residual toxicity at a specific concentration, the higher concentrations are not explored.

### 4.10. Animal Use Statement

Research was conducted under an IACUC approved protocol in compliance with the Animal Welfare Act, PHS Policy, and other Federal statutes and regulations relating to animals and experiments involving animals. The facility where this research was conducted is accredited by the Association for the Assessment and Accreditation of Laboratory Animal Care, International and adheres to principles stated in the Guide for the Care and Use of Laboratory Animals, National Research Council, 2011.

## Figures and Tables

**Table 1:** Potency ED_50_ values (**bold**) and 95% confidence limits for ciBoNT HP and BoNT Hc vaccines in monovalent and multivalent formulations at T = 0 months. Recombinant ciBoNT/B HP was unavailable for this study.

Challenge Toxin		BoNT/A1	BoNT/B1	BoNT/C	BoNT/E3	BoNT/F1
**ciBoNT HP formulation**		**A1**	**B1**	**C1**	**E1**	**F1**
monovalent ACEF	**0 months**	**8 ng**	**-----**	**11 ng**	**49 ng**	**11 ng**
		5–13 ng		6–19 ng	29–86 ng	3–98 ng
	**0 months**	**7 ng**	**-----**	**11 ng**	**139 ng**	**10 ng**
		4–12 ng		3–21 ng	29 ng–1.2 µg	4–15 ng
**BoNT Hc formulation**		**A1**	**B1**	**C1**	**E1**	**F1**
monovalent ABCEF	**0 months**	**34 ng**	**57 ng**	**113 ng**	**222 ng**	**163 ng**
		17–59 ng	33–98 ng	57–212 ng	95–557 ng	89–288 ng
	**0 months**	**22 ng**	**72 ng**	**220 ng**	**1.53 µg**	**626 ng**
		5–44 ng	28–153 ng	122–395 ng	580 ng–18 µg	312 ng–1.8 µg

**Table 2:** Stability potency ED_50_ values (**bold**) and 95% confidence limits for ciBoNT HP and BoNT Hc vaccines in monovalent and multivalent formulations at T = 6 months. Results are from studies assessing vaccines after initial formulation and storage for 6 months at 2–8 °C. Recombinant ciBoNT/B HP was unavailable for this study.

Challenge Toxin		BoNT/A1	BoNT/B1	BoNT/C	BoNT/E3	BoNT/F1
**ciBoNT HP formulation**		**A1**	**B1**	**C1**	**E1**	**F1**
monovalent ACEF	**6 months**	**12 ng**	**-----**	**21 ng**	**132 ng**	**12 ng**
		0.7–60 ng		13–33 ng	68–279 ng	6–22 ng
	**6 months**	**6 ng**	**-----**	**21 ng**	**129 ng**	**20 ng**
		3–10 ng		0.1–282 ng	74–231 ng	----
**BoNT Hc formulation**		**A1**	**B1**	**C1**	**E1**	**F1**
monovalent ABCEF	**6 months**	**19 ng**	**50 ng**	**82 ng**	**201 ng**	**126 ng**
		11–30 ng	19–98 ng	49–133 ng	89–456 ng	54–266 ng
	**6 months**	**14 ng**	**54 ng**	**71 ng**	**969 µg**	**364 ng**
		12–33 ng	20–110 ng	29–142 ng	529 ng–2.5 µg	198–738 ng

**Table 3:** Amino acid sequence identity comparisons of ciBoNT HP and BoNT Hc vaccines with homologous and heterologous toxins. * BoNT/B4 is produced in a nonproteolytic Group II *C. botulinum* strain; ** BoNT/E4 produced in *C. butyricum*; *** BoNT/F7 produced in *C. baratii.*

Vaccine Sequence vs.:	BoNT HP	BoNT Hc
BoNT/A1	100%	100%
BoNT/A2	89.9%	87.0%
BoNT/A3	84.6%	86.5%
BoNT/B1	100%	100%
BoNT/B2	95.6%	91.6%
BoNT/B4 *	93.2%	89.5%
BoNT/C1	100%	100%
BoNT/CD	75.9%	41.2%
BoNT/DC	64.7%	75.8%
BoNT/E1	99.8%	99.8%
BoNT/E3	98.1%	99.6%
BoNT/E4 **	97.2%	97.3%
BoNT/F1	100%	100%
BoNT/F7 ***	68.6%	

**Table 4:** Potency ED_50_ values (**bold**) and 95% confidence limits for ciBoNT/A HP, ciBoNT/B HP, ciBoNT/C HP, and ciBoNT/E HP vaccines and BoNT A Hc, BoNT/B Hc, BoNT/C Hc, and BoNT/E Hc vaccines against the specified toxin subtypes. Data for ciBoNT/A HP and BoNT/A Hc taken from [25]. Animals were vaccinated once for all potencies except where noted.

**A**	**Challenge Toxin:**	**BoNT A1**	**BoNT A2**	**BoNT A3**
	ciBoNT/A1 HP	**18 ng**	**132 ng**	**144 ng**
		14–23 ng	75–220 ng	2 ng–1.2 µg
	BoNT/A1 Hc	**52 ng**	**5.9 µg**	**18 µg**
		28–86 ng	3.7–11 µg	4.7–36 µg
**B**	**Challenge Toxin:**	**BoNT B1**	**BoNT B2**	**BoNT B4**
	ciBoNT/B1 HP	19 ng	67 ng	32 ng
		-----	-----	19–56 ng
	BoNT/B1 Hc:	**33 ng**	**24 µg**	**77 µg**
		15–57 ng	-----	-----
**C**	**Challenge Toxin:**	**BoNT C1**	**BoNT CD**	**BoNT DC**
	ciBoNT/C1 HP	**15 ng**	**27 ng**	*
		8–24 ng	17–85 ng	
	BoNT/C1 Hc	**101 ng**	*	*
		48–192 ng		
**D**	**Challenge Toxin:**	**BoNT E1**	**BoNT E3**	**BoNT E4**
	ciBoNT/E1 HP (2X)	**12 ng**	**4 ng**	**22 ng**
		1–21 ng	-----	9–39 ng
	ciBoNT/E1 HP (1X)	**95 ng**	**62 ng**	**160 ng**
		42–190 ng	23–129 ng	31–797 ng
	BoNT/E1 Hc (2X)	**1.13 µg**	**2.42 µg**	**>10 µg**
		104 ng–13.4 µg	105 ng -->	-----

* Unable to calculate an ED_50_ due to too few survivors.

**Table 5:** Comparative efficacy studies of protection after multiple vaccinations with ciBoNT HP and BoNT/Hc vaccines and challenge with homologous and heterologous toxin subtypes. All animals were vaccinated with 1 µg of vaccine and challenged with 1,000 mouse IP LD_50_ of toxin. The ciBoNT/A1 HP and /A1 Hc efficacy studies were previously published [25].

**A**	**ciBoNT/A HP**				**BoNT/A Hc**			
	**1X**		**2X**		**1X**		**2X**	
BoNT/A1	10/10		10/10		8/10		10/10	
BoNT/A2	5/10		10/10		2/10		9/10	
BoNT/A3	3/10		10/10		0/10		9/10	
**B**	**ciBoNT/B HP**				**BoNT/B Hc**			
	**1X**	**2X**		**3X**	**1X**	**2X**		**3X**
BoNT/B1	10/10	10/10		10/10	10/10	10/10		10/10
BoNT/B2	10/10	10/10		9/9	0/10	7/10		9/10
BoNT/B4	10/10	10/10		10/10	1/10	7/10		7/10
**C**	**ciBoNT/C HP**				**BoNT/C Hc**			
	**1X**	**2X**		**3X**	**1X**	**2X**		**3X**
BoNT/C1	10/10	10/10		10/10	10/10	10/10		10/10
BoNT/CD	10/10	10/10		10/10	2/10	2/10		3/10
BoNT/DC	0/10	9/10		9/10	0/10	6/10		7/10

**Table 6:** Mouse toxicity bioassay. Mice were given the described dose of the ciBoNT vaccine antigens IP and observed for 15 days.

Vaccine Antigen	Dose	Survival
ciBoNT/A1	50 µg	5/5
ciBoNT/B1	25 µg	5/5
ciBoNT/C1	25 µg	3/5
ciBoNT/E1	5 µg	3/5
ciBoNT/F1	25 µg	5/5

**Table 7:** BoNT subtypes, strains, toxicities, and origins for the toxins.

Toxin Subtype	Strain	Lot #	Toxicity (LD_50_/mL)	Produced by:
BoNT/A1	Hall	A111104-01	5.3 × 10^7^	Metabiologics
BoNT/A2	FRI-honey	A021705.01	4.1 × 10^7^	Metabiologics
BoNT/A3	Loch Maree	AU051405-01	1.9 × 10^5^	USAMRIID
BoNT/B1	Okra	B020305-01	2.8 × 10^7^	Metabiologics
BoNT/B2	213B	BU051405-01	2.6 × 10^5^	USAMRIID
BoNT/B4	Eklund 17B	17B101005	3.7 × 10^8^	Metabiologics
BoNT/C1	Brazil	C072206-01	6.3 × 10^6^	Metabiologics
BoNT/CD	003-9	LKF7442	6.7 × 10^7^	Waco
BoNT/DC	VPI 5995	D010604-01	5.0 × 10^7^	Metabiologics
BoNT/E1	German sprats	CER5845	2.1 × 10^6^	Waco
BoNT/E3	Alaska	E062205-01	4.0 × 10^7^	Metabiologics
BoNT/E4	BL5262	EU033107	1.6 × 10^6^	USAMRIID
BoNT/F1	Langeland	F030501-01	6.0 × 10^6^	Metabiologics
BoNT/F7	Sullivan	FU032610	7.5 × 10^4^	USAMRIID
